# Placental Expression of miR-517a/b and miR-517c Contributes to Trophoblast Dysfunction and Preeclampsia

**DOI:** 10.1371/journal.pone.0122707

**Published:** 2015-03-23

**Authors:** Lauren Anton, Anthony O. Olarerin-George, John B. Hogenesch, Michal A. Elovitz

**Affiliations:** 1 Maternal and Child Health Research Program, Department of Obstetrics and Gynecology, Perelman School of Medicine at the University of Pennsylvania, Philadelphia, Pennsylvania, United States of America; 2 Genomics and Computational Biology Graduate Group, Perelman School of Medicine at the University of Pennsylvania, Philadelphia, Pennsylvania, United States of America; 3 Department of Pharmacology and the Institute for Translational Medicine and Therapeutics, Perelman School of Medicine at the University of Pennsylvania, Philadelphia, Pennsylvania, United States of America; Chinese Academy of Sciences, CHINA

## Abstract

Preeclampsia is a pregnancy specific hypertensive disease that confers significant maternal and fetal risks. While the exact pathophysiology of preeclampsia is unknown, it is widely accepted that placental dysfunction is mechanistically involved. Recent studies reported aberrant expression of placenta-specific microRNAs (miRNAs) in preeclampsia including miR-517a/b and miR-517c. Using placental biopsies from a preeclampsia case-control study, we found increased expression of miR-517a/b in term and preterm preeclampsia vs controls, while, miR-517c was increased only in preterm preeclampsia vs controls. To determine if miR-517a/b and miR-517c are regulated by hypoxia, we treated first trimester primary extravillous trophoblast cells (EVTs) with a hypoxia mimetic and found both were induced. To test for a mechanistic role in placental function, we overexpressed miR-517a/b or miR-517c in EVTs which resulted in decreased trophoblast invasion. Additionally, we found that miR-517a/b and miR-517c overexpression increased expression of the anti-angiogenic protein, sFLT1. The regulation of sFLT1 is mostly unknown, however, TNFSF15, a cytokine involved in FLT1 splicing, was also increased by miR-517a/b and miR-517c in EVTs. In summary, we demonstrate that miR-517a/b and miR-517c contribute to the development of preeclampsia and suggest that these miRNAs play a critical role in regulating trophoblast and placental function.

## Introduction

Preeclampsia affects 5–10% of pregnancies worldwide and continues to be a major contributor to maternal morbidity and mortality. Severe preeclampsia, often occurring earlier than 37 weeks, is associated with maternal morbidity and adverse neonatal and fetal outcomes including perinatal death, preterm birth, small for gestational age infants, and intrauterine growth restriction [1–3]. Despite the extensive research investigating the pathogenesis of preeclampsia, the primary causes of this disease are unknown. However, several theories have sought to explain the development of preeclampsia, including abnormal placental implantation and function [4], imbalance of angiogenic factors [5, 6], changes in placental oxygen tension [7] and alterations in the placental and maternal immune response [8]. Although symptoms of preeclampsia can exist after delivery, clinically, delivery of the placenta remains the only treatment suggesting that the origins of this disease may lie within the placenta.

Recent literature suggests that poor placental development and abnormal placentation early in pregnancy is mechanistically involved in the development of disease. Poor placentation, which is characterized by shallow trophoblast invasion into the maternal uterine spiral arteries, results in narrow vessel diameter, decreased blood flow and, ultimately, poor uteroplacental perfusion [9]. Consequently, the placenta becomes increasingly ischemic/hypoxic as gestation progresses resulting in altered placental secretion/expression of many factors. These include soluble fms-like tyrosine kinase 1 (sFLT1, soluble VEGF receptor) [5, 10, 11] and inflammatory cytokines [12, 13], among many others, which are known to be associated with preeclampsia pathogenesis. sFLT1 is a splice variant of the membrane bound VEGF receptor 1 (VEGFR1, FLT1) and is classified as an anti-angiogenic protein by acting as a decoy receptor and blocking the downstream signaling of VEGF and PLGF. While the mechanisms regulating VEGFR1 splicing are largely unknown, a recent study has indicated that the tumor necrosis factor super family 15 (TNFSF15) signaling pathway may be involved [14]. However, not much is known about the presence, function or regulation of TNFSF15 in the placenta.

To identify the molecular mechanisms contributing to the placental dysfunction seen in preeclampsia, many studies have looked at differential gene expression between preeclamptic and normal placentas [15, 16]. These studies provide clear evidence that changes in placental gene expression contribute to the development of preeclampsia. However, the regulatory mechanisms controlling these gene changes remain largely unknown.

In recent years, microRNAs (miRNAs) have emerged as important regulators of gene expression in many disease related pathologies. miRNAs are small, non-coding RNAs that regulate gene expression primarily at the post-transcriptional level. miRNAs function by binding to the 3’-untranslated region (UTR) of target genes resulting in post-transcriptional repression [17]. As this binding can occur with partial complementarity, one miRNA has the ability to bind to multiple genes (even hundreds) and one gene can be regulated by many miRNAs [18].

Increasing evidence suggests that miRNAs are important regulators of placental development and function. Many studies identified placenta specific miRNAs [18, 19] including one study that found more than 600 miRNAs in healthy term placenta [20]. The most highly expressed placental miRNAs were found to be encoded by the primate specific C19MC- cluster of miRNAs located on chromosome 19 [21]. Encoding 54 predicted miRNAs [22], C19MC is the largest human miRNA gene cluster and is almost exclusively placental specific [23]. miRNAs in the C19MC cluster comprise the majority of human term trophoblast miRNAs which includes the miR-517 family of miRNAs [23, 24]. The miR-517 family consists of three isoforms, miR-517a, miR-517b, and miR-517c [25]. However, sequence data from miRBase [26], a central repository for published miRNA sequences, lists an identical sequence for miR-517a (hsa-miR-517a-3p, accession # MIMAT0002852) and miR-517b (hsa-miR-517b-3p, accession # MIMAT0002857) indicating that miR-517a/b have identical target genes and functions. Due to their sequence homology, miR-517a and miR-517b will henceforth be referred to as miR-517a/b. Recent studies have reported aberrant expression of placenta-specific miRNAs in preeclamptic pregnancies [27–29]. Several studies used arrays to investigate differential expression of miRNAs in preeclamptic placentas and identified increased expression of miR-517c [30] and miR-517* (miR-517-5p) [28]. The expression of miR-517a/b in preeclamptic placentas has not been investigated. While the functions of miR-517a/b and miR-517c remain largely unknown, their placental expression suggests a role in the regulation of altered placental and trophoblast function including abnormalities in trophoblast invasion known to be associated with preeclampsia.

Here we investigate whether altered expression of miR-517a/b and miR-517c result in trophoblast dysfunction that contributes to the development of preeclampsia. To address this, first we used an existing case-control study to determine 1) if miR-517a/b and miR-517c are altered in preeclamptic placentas and 2) if these expression changes were associated with disease severity. Second, we used primary first trimester extravillous trophoblast (EVT) cells to investigate if miR-517a/b and miR-517c are regulated by hypoxia, a known contributor to the development of preeclampsia. Lastly, using primary EVTs, we tested whether increased expression of miR-517a/b and miR-517c altered trophoblast invasion, TNFSF15 expression and the release of angiogenic factors—sFLT1, VEGF and PLGF.

## Materials and Methods

### Ethics Statement

The Preeclampsia: Mechanisms and Consequences case-control study was performed with approval from the University of Pennsylvania Institutional Review Board. Written informed consent was obtained from all study participants prior to sample collection. Extravillous trophoblast cells lines were isolated from de-identified first trimester placental tissues and were, therefore, exempt from IRB approval (University of Pennsylvania Institutional Review Board (IRB #7)).

### Case-control study

A case-control study (Preeclampsia: Mechanisms and Consequences) was performed between March 2005 and October 2009 at the Hospital of the University of Pennsylvania. Controls were defined as women without hypertension-related complications that presented for delivery at term (≥37 gestational weeks). Cases were identified based on pre-specified maternal criteria according to standard American College of Obstetricians and Gynecologists (ACOG) [31–33]. Based on these pre-specified criteria, case eligibility was determined at the time of enrollment by the study investigators and not by the treating physician. Demographic variables were compared between all cases and controls and between term and preterm cases using t-tests. Immediately after delivery, placental biopsies were taken from cases and controls. After removal of the fetal membranes, four full-thickness (0.5 inch in diameter) random biopsies were taken by a trained research coordinator. The tissues were washed in sterile saline and immediately frozen in liquid nitrogen and stored at -80°C until use. Placental expression of miR-517a/b and miR-517c was assessed by qPCR. Clinical information including race, BMI at first prenatal visit, maternal age and other maternal and prenatal factors were abstracted from the patient’s medical record.

### Cell Culture

Primary extravillous trophoblast cells (EVT) were isolated from first trimester villous tissue using a protocol that has been established and well documented by Graham *et al*. [34–36]. Briefly, finely minced chorionic villi collected from de-identified elective first trimester pregnancy termination tissues (less than 12 weeks) were cultured at 37^°^C in RPMI 1640 medium containing 20% charcoal-stripped (steroid-free) fetal bovine serum (FBS). EVT cells, which outgrow from attached villous fragments, were separated from villous tissue during washing and passaging of the cells. The isolated EVT cells were maintained in RPMI 1640 medium containing 20% FBS and 1% penicillin (100 U/mL)/ streptomycin (100 U/mL) solution. The EVT cells used in our experiments were characterized by immunostaining for trophoblast cell markers, cytokeratin-7, 8 and 18 and integrin alpha-1 [35, 37–39]. These results are similar to those obtained by other investigators using the same EVT isolation methods and confirm the purity of the EVT cell preparations [34, 36].

### EVT Cobalt Chloride Treatment

EVTs were plated at 2.5 x 10^5^ cells/well in 24-well plates. Forty eight hours later, the cells were treated with 0–100 uM of CoCl_2,_ (Sigma, St. Louis, MO) a hypoxia mimetic [40, 41], or vehicle control (water) dissolved in RPMI 1640 for 6 hours (n = 4). After treatment, the media was aspirated, the cells were washed with PBS and 500uL of Trizol (Invitrogen, Life Technologies, Grand Island, NY) was added to each well for future miRNA extraction.

### EVT Cell Transfection

EVT cells were plated at 1x10^5^ cells/well in 6-well plates in antibiotic-free 1640 RPMI media containing 20% FBS. The next day, the cells were transfected with miRNA mimics (40 nM) or miRNA inhibitors (40nM). Hsa-miR-517a/b, hsa-miR-517c and miR-negative (non-targeting control) miRNA mimics and anti-miR-517a/b, anti-miR-517c and anti-miR-negative control inhibitors were purchased from Ambion (Life Technologies). Lipofectamine RNAiMAX (Invitrogen) was used for the transfection of the miRNA mimics and inhibitors according to the manufacturers’ protocol. Cells were transfected for 48 to 72 hours and maintained under normal growth conditions. Cells were then used for invasion assays or QPCR measurement of target genes or media was collected for angiogenic growth factor measurement by ELISA.

### miRNA Isolation from Placental Tissue and EVT Cells

Approximately 25 mg of placenta tissue per sample was homogenized in 500uL of Trizol with 5mm steel beads using a TissueLyser (Qiagen, Valencia, CA) set at 30 Hz for 3 mins (2 times). Placental tissue and EVT cells (with or without miRNA transfection) in Trizol underwent phenol-chloroform extraction and the resulting aqueous phase was further column purified with the miRNeasy kit (Qiagen) according to the manufacturer’s protocol for total RNA isolation including small RNAs. RNA concentration was determined via a NanoDrop 2000 Spectrophotometer (Nanodrop Rockland, DE) prior to the generation of cDNA.

### cDNA generation and qPCR

cDNA was generated from 1μg of isolated RNA from either placental tissue or EVT cells (with or without transfection) using the miScript Reverse Transcription II kit (Qiagen) or high capacity cDNA reverse transcription kit (Applied Biosystems, Life Technologies) for SYBR Green or TaqMan primers, respectively. qPCR was performed on the 7900HT Real-Time PCR System (Applied Biosystems) using the miScript SYBR Green PCR kit (Qiagen) or Taqman Universal PCR Master Mix (Applied Biosystems) according to the manufacturers’ protocols. The ΔΔCT (SYBR Green PCR) or standard curve (TaqMan PCR) method was used for relative expression quantification using the RQ manager software v2.4 (Applied Biosystems). For SYBR Green PCR, the endogenous reference genes SNORD25 and RNU6B were used for miRNA quantification from placental tissues and EVT cells, respectively. All primer sets were purchased from Qiagen: hsa-miR-517a/b (MS00004459), hsa-miR-517c (MS00009954), SNORD25 (MS00014007) and RNU6B (MS0001400). For TaqMan PCR, the relative abundance of the target of interest was divided by the relative abundance of 18S in each sample to generate a standardized abundance for the target transcript of interest. All primer sets were purchased from Applied Biosystems: TNFSF15, JMJD6, U2AF2, JMJD8 and 18S.

### Western Blots

EVTs were plated at 1x10^5^ cells/well in a 6-well plate in 2mL of media (1640 RPMI + 20% FBS). 48hrs later, the cells were treated with 100uL of a CoCl_2_ solution in RPMI for the indicated times. The final concentration of CoCl_2_ in each well was 100 uM. The cells were rinsed with ice cold PBS at the time of harvesting and whole cell protein lysates were extracted with ice-cold RIPA buffer. Protein concentrations were estimated with the DC protein assay (BioRad, Hercules, CA) according to the manufacturer’s protocol. 20ug of each protein lysate was resolved via SDS-PAGE and transferred to PVDF membranes. The membranes were blocked for 1hr at room temperature with blocking buffer (5% milk, 0.5% Tween20, 1X Tris-buffered saline) and then probed with HIF1α (Cell Signaling, Beverly, MA; 3716S) or alpha tubulin (Abcam, Cambridge, MA; ab7291) primary antibodies in blocking buffer at 4°C overnight. Membranes were rinsed twice each with wash buffer (0.5% Tween20, 1X Tris-buffered saline) then blocking buffer, then probed with anti-rabbit or anti-mouse IgG HPR-linked secondary antibodies (GE Healthcare, Piscataway, NJ) in blocking buffer for 30 minutes at room temperature. Membranes were rinsed 4 times in wash buffer for 10–15 minutes each. Chemiluminesce ECL reagent (Perkin Elmer, Waltham, MA) was added to the membranes according to the manufacturer’s instructions. Membranes were then exposed to X-ray film.

### Matrigel Invasion Assay

The invasiveness of primary EVT cells through an extracellular matrix was measured using a commercially available cell invasion assay kit (Chemicon, Temecula, CA). Briefly, EVT cells were 1) treated with CoCl_2_ (100uM) for 24 hours prior to being used for the invasion assay and for an additional 72 hours in the media in the top chamber during the invasion assay or 2) transfected with miR-negative control, miR-517a/b and miR-517c mimics or inhibitors (as described above) for 48 hours prior to the start of the invasion assay. The transfected EVT cells (300ul of 1x10^6^ cells/ml suspension) were then plated onto ECMatrix gel-coated cell culture inserts (8 μm pore size) in 24 well plates (n = 3 per treatment group). After 72 hours, the non-invading cells and the ECMatrix gel from the upper surface of the inserts was removed using a cotton-tipped swab. Invasive cells on the lower surface of the membrane were stained with 0.2% crystal violet for 20 minutes. The membranes were mounted onto microscope slides. Stained cells from five random microscope fields at 20X magnification were photographed, counted and analyzed. Data from experiments measuring EVT invasion are expressed as a percent of control.

### Angiogenic Growth Factor ELISAs

EVTs were cultured and transfected as stated above. sFLT1, VEGF and PLGF were measured in EVT cell culture media after 48 hours of transfection with miR-negative, miR-517a/b or miR-517c mimics (n = 4). The expression of sFLT1, VEGF and PLGF were measured by ligand specific commercially available ELISA kits that utilize a quantitative sandwich enzyme immunoassay technique using regents from R&D Systems (Minneapolis, MN).

### Lactate Dehydrogenase (LDH) Cytotoxicity Assay

The viability of EVT cells (n = 3), after transfection with miR-517a/b, miR-517c and miR-negative, was determined by measuring LDH leakage into the medium using the CytoTox 96 Non-Radioactive Cytotoxicity Assay (Promega Corp, Madison, WI). After collecting cell culture medium, the EVTs were lysed with RIPA Buffer containing protease and phosphatase inhibitors (Sigma, St. Louis, MO). Released LDH was measured with a coupled enzymatic assay that results in the conversion of a tetrazolium salt into a red formazan product. The amount of color formed is proportional to the number of lysed cells. The enzymatic reaction was measured spectrophotometrically by absorbance readings at 490 nm. The percentage of cytotoxicity was calculated as experimental LDH (cell culture media)/maximum release of LDH (cell lysates).

### Statistical Analysis

Statistical analyses for the in vitro studies were performed for all experiments with the GraphPad Prism Software (Version 4.0, San Diego, CA). For data that were normally distributed, t-tests or one-way analysis of variance (ANOVA) was used. If statistical significance was reached (p<0.05), then pair-wise comparison with a Student-Newman-Keuls (SNK) test was performed. If data were not normally distributed then the non-parametric Mann-Whitney test was used.

## Results

### Clinical and demographic characteristics of case-control study

We investigated the association between preeclampsia and the expression of miR-517a/b and miR-517c in placentas from our case-control study. Clinical and demographic characteristics of the study groups are shown in Table 1. There was a statistically significant decrease in mean gestational age at delivery and mean fetal weight in cases versus controls (p<0.05) and in preterm cases versus term cases (p<0.0001). All other parameters including maternal age, race, body mass index and fetal gender were not statistically different.

**Table 1:** Clinical and demographic characteristics of Preeclampsia case-control study.

	Case—Control Analysis		Case—Case Analysis	
	Control (n = 14)	Preeclampsia (n = 31)	Term PE (n = 18)	Preterm PE (n = 13)
Mean maternal age (years)	27.0 ± 7.2	28.1 ± 7.7	28.3 ± 8.2	27.8 ± 7.3
Mean gestational age at delivery (weeks)	39.2 ± 1.2	35.7 ± 4.6*	38.8 ± 1.0	31.4 ± 4.0**
Race, n (%)				
African American	11 (78.6)	25 (80.6)	14 (77.8)	11 (84.6)
Other	3 (21.4)	6 (19.4)	4 (22.2)	2 (15.4)
Mean Body Mass Index (BMI) (lbs/inches^2^)	30.1 ± 8.2^‡^	30.3 ± 7.5^‡^	31.7 ± 6.8^‡^	28.2 ± 8.2^‡^
Mean Fetal Weight (grams)	3272 ± 467	2630 ± 962*	3225 ± 534	1807 ± 804*
Fetal Gender, n (%)				
Male	5 (35.7)	17 (54.8)	7 (38.9)	10 (76.9)
Female	9 (64.3)	14 (45.2)	11 (61.1)	3 (23.1)

Data expressed as Mean ± SD.

^‡^BMI at first prenatal visit with an average gestational age of 18 weeks (controls), 15 weeks (cases), 14 weeks (term cases) or 17 weeks (preterm cases).

*p<0.05 versus Controls,

**p<0.0001 versus Term Cases

### miR-517a/b and miR-517c expression are up-regulated in preeclamptic placentas

**Fig 1:**
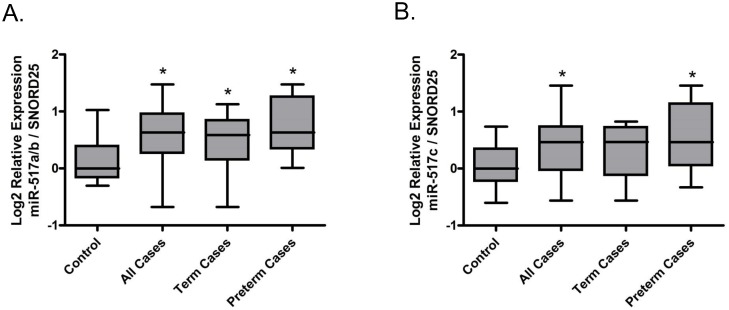
Placental miR-517a/b and miR-517c expression is upregulated in preeclampsia. Boxplots of miR-517a/b (**A**) and miR-517c (**B**) expression in human placentas from a preeclampsia case-control study. Case cohort (case-all) samples were divided into preterm (case-preterm) and term (case-term) subgroups. All comparisons are relative to the control group. *p<0.05

miR-517a/b (p = 0.0085, Fig. 1A) and miR-517c (p = 0.0043, Fig. 1B) were significantly up-regulated in preeclamptic placentas relative to control placentas. Premature delivery is often necessary in severe preeclampsia due to fetal and maternal health complications. Hence, preterm status in our cohort served as an indicator of disease severity. We stratified the preeclamptic placental samples into term and preterm based on gestational age at delivery and compared miRNA expression to that of the control (normal term deliveries). miR-517a/b was significantly up-regulated in both term (p = 0.0426) and preterm (p = 0.0060) preeclamptic placentas relative to the control (Fig. 1A) while miR-517c was significantly up-regulated in the preterm cases (p = 0.0098) but not in the term cases (p = 0.0547) (Fig. 1B). These results suggest that placental expression of miR-517a/b and miR-517c may be further correlated with the severity of preeclampsia.

### miR-517a/b and miR-517c expression is regulated by hypoxia

**Fig 2:**
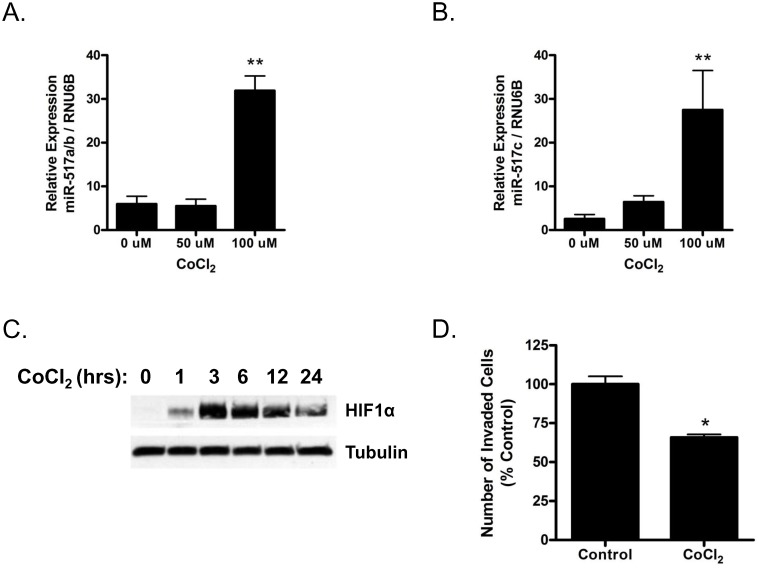
Hypoxia upregulates trophoblast miR-517a/b and miR-517c expression. miR-517a/b (**A**) and miR-517c (**B**) expression in EVT cells treated with the indicated doses (0–100 μM) of CoCl_2_ for 6hrs. Time course of HIF1α protein (C) expression in EVTs treated with CoCl_2_ (100 μM). (D) Invasion assay of EVTs treated with 100 μM CoCl_2_. Values are mean ± SEM. n = 4 (A and B), n = 3 (D) *p<0.05, **p < 0.01.

Studies suggest that preeclampsia is associated with abnormal placental hypoxia early in pregnancy [42, 43]. Therefore, in order to determine if miR-517a/b and miR-517c expression is regulated by hypoxia, we treated first trimester primary EVT cells with a hypoxia mimetic, cobalt chloride (CoCl_2_) [40, 41]. CoCl_2_ induced miR-517a/b (p = 0.0003, Fig. 2A) and miR-517c (p = 0.0027, Fig. 2B) expression. miR-517a/b and miR-517c expression was significantly induced in EVTs treated with 100 uM of CoCl_2_ for 6hrs (miR-517a/b: p<0.0001; Fig. 2A and miR-517c: p<0.01, Fig. 2B). To confirm that CoCl_2_ was mimicking hypoxia in the EVTs, we measured HIF1α protein expression in cells exposed to CoCl_2_ over the course of 24 hours (Fig. 2C). Expression of HIF1α protein was evident as early as one hour after treatment, peaked at three hours (a time period prior to the observed increase in miR-517), and remained high as long as 24 hours post treatment. As hypoxia is a known regulator of trophoblast function, we assessed if the hypoxia mimetic, CoCl_2,_ altered trophoblast invasion. Indeed, treatment of EVTs with CoCl_2_ decreased EVT invasion by 40% relative to the negative control (p = 0.014; Fig. 2D). These results suggest that CoCl_2_ induces hypoxia in EVTs, which in turn activates HIF1α protein and miR-517a/b and miR-517c expression.

### miR-517a/b and miR-517c decrease trophoblast invasion

**Fig 3:**
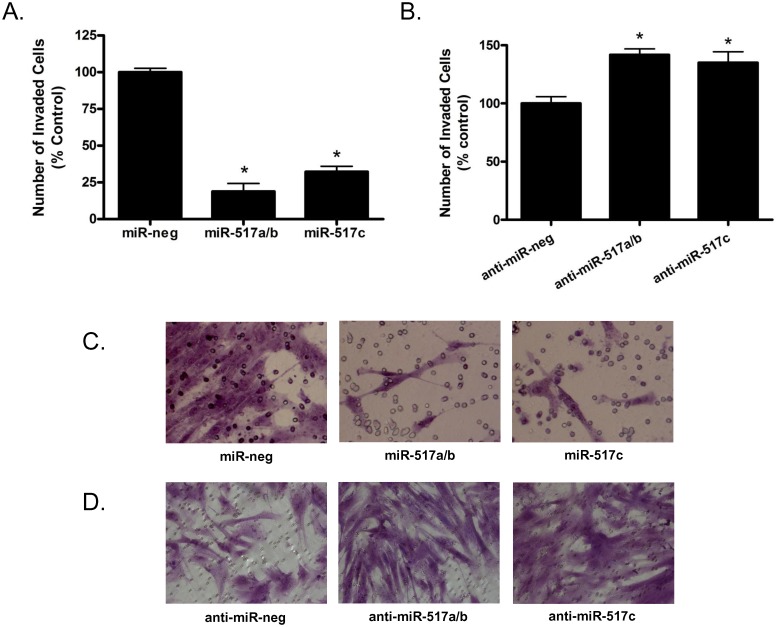
miR-517a/b and miR-517c regulates trophoblast invasion. Bar graph results from invasion assays of EVT cells transfected with (**A**) miR-517a/b/c mimics or or (**B**) miR-517a/b/c inhibitors. Associated light microscopy photographs of invaded cells transfected with (**C**) miR-517a/b/c mimics or (**D**) miR-517a/b/c inhibitors. Values in (**A) and (B**) are expressed as a percent of control (miR-negative or anti-miR-negative transfected cells). n = 3. *p<0.001

Given the results of our clinical studies showing that the expression of miR-517a/b and miR-517c are increased in preeclamptic placentas and that these miRNAs can be induced by hypoxia, we sought to determine if these miRNAs altered placental function, specifically EVT invasion, a process known to be abnormal in preeclampsia. Invasion was decreased (p<0.0001) in EVTs transfected with miR-517a/b and miR-517c (Figs. 3A and 3C). miR-517a/b decreased invasion by 80.9% (p = 0.0002) and miR-517c decreased invasion by 67.9% (p = 0.0001) compared to EVTs transfected with a miR-negative control. EVTs were also transfected with inhibitors of miR-517a/b and miR-517c in order to silence endogenous miR-517 levels. miR-517a/b inhibition increased EVT invasion by 41.8% (p = 0.0057) and miR-517c inhibition increased invasion by 35% (p = 0.0337) compared to anti-miR-negative control transfected cells (Figs. 3B and 3D). Additionally, transfection of EVTs with miR-517a/b and miR-517c had no effect on levels of LDH released into the media (miR-517a/b: 0.96% cytotoxicity miR-neg vs 1.05% cytotoxicity miR-517a/b, miR-517c: 0.96% cytotoxicity miR-neg vs 1.03% cytotoxicity miR-517c). These results indicate that the decreased invasion observed in EVTs was not a result of cell death caused directly by the transfection of miR-517a/b or miR-517c but was instead a regulated response by their downstream targets.

### miR-517a/b and miR-517c increase sFLT1 expression

**Fig 4:**
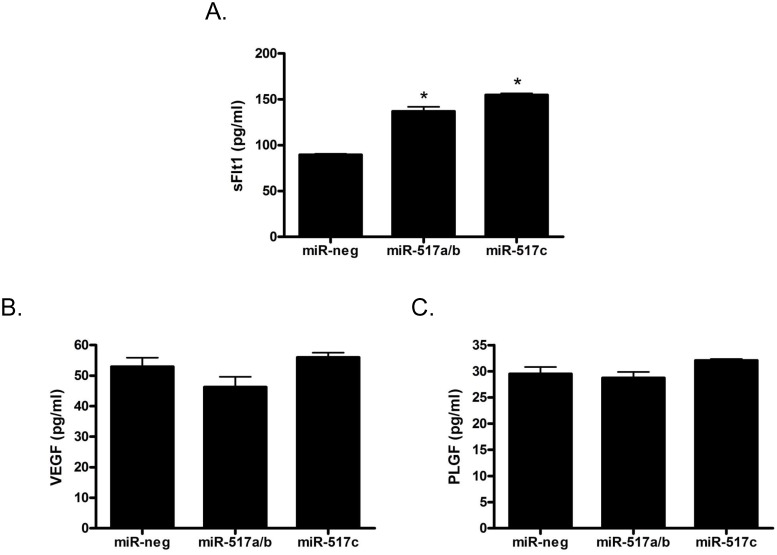
miR-517a/b and miR-517c increases the release of sFLT1 from trophoblast cells. sFtl1 (**A**), VEGF (**B**) and PLGF (**C**) levels in cell culture media after extravillous trophoblast cell transfection with miR-negative control, miR-517a/b and miR-517c. VEGF and PLGF levels were unchanged by miR-517a and miR-517c transfection. Values are mean ± SEM. n = 4 *p<0.001.

Previous studies showed that an imbalance of placental angiogenic factors, specifically sFLT1, VEGF and PLGF; contribute significantly to the development of preeclampsia. Thus, we investigated if increased amounts of miR-517a/b and miR-517c, as was seen in our preeclamptic placentas, could alter the release of angiogenic factors from primary first trimester EVT cells. EVTs transfected with miR-517a/b (p<0.001) and miR-517c (p<0.001) showed a significant increase in the release of sFLT1 (Fig. 4A). Further, there were no significant differences observed in VEGF or PLGF in EVTs transfected with miR-517a/b or miR-517c (Figs. 4B and 4C).

### miR-517a/b and miR-517c increase TNFSF15 expression

**Fig 5:**
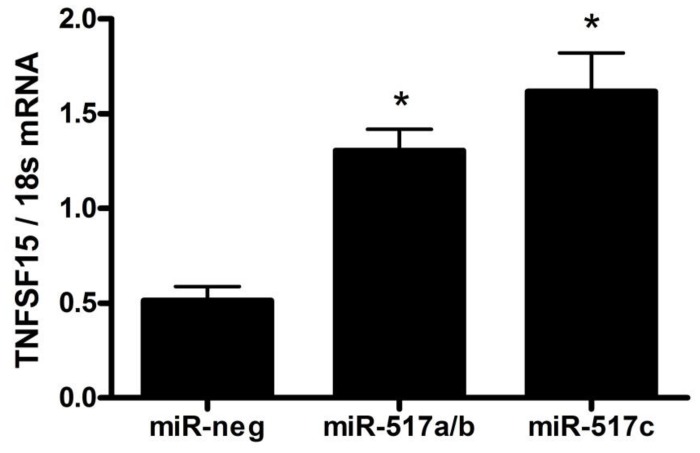
miR-517a/b and miR-517c increases TNFSF15 expression in trophoblast cells. TNFSF15 expression in extravillous trophoblast cells transfected with miR-negative control, miR-517a/b and miR-517c. Values are mean ± SEM. n = 5 *p<0.001.

Due to the finding that miR-517a/b and miR-517c overexpression resulted in an increase in sFLT1, we further investigated other potential upstream regulators linking miR-517a/b and miR-517c to alternative sFLT1 splicing. As the tumor necrosis factor super family 15 (TNFSF15) pathway has recently been implicated in sFLT1 regulation [14, 44] we focused on the effects of miR-517a/b and miR-517c on TNFSF15, Jumonji domain-containing protein 6 (JMJD6) and the splicing factor U2 small nuclear RNA auxiliary factor 2 (U2AF2). TNFSF15 expression was significantly increased by both miR-517a/b (p = 0.0009) and miR-517c (p = 0.0024) (Fig. 5). However, JMJD6 and U2AF2 were not changed by the overexpression of either of these miRNAs (Table 2). Additionally, we included JMJD8, a known predicted target of miR-517a/b and miR-517c, as a positive control which was, as expected, significantly decreased with miR-517a/b (p = 0.0186) and miR-517c (p = 0.0036) transfection (Table 2).

**Table 2:** mRNA expression levels of TNFSF15 associated pathway genes involved in sFLT1 regulation in first trimester extravillious trophoblast cells transfected with miR-517a/b and miR-517c.

	miR-517a/b		miR-517c	
Gene	Fold Change	P value	Fold Change	P value
JMJD6	1.18	0.0833	1.16	0.1214
U2AF2	1.04	0.7403	-1.02	0.8909
JMJD8	-1.36	0.0186	-1.63	0.0036

## Discussion

Here, we report that miR-517a/b and miR-517c may contribute to the development of preeclampsia by altering first trimester extravillous trophoblast function. First, we demonstrated that an increased placental expression of miR-517a/b and miR-517c at term is associated with preeclampsia suggesting a role for these miRNAs in placental function. Second, in first trimester trophoblasts we found that hypoxia results in increased miR-517a/b and miR-517c expression indicating that the abnormally hypoxic placental environment, known to be associated with preeclampsia, could induce these miRNAs. Third, in first trimester EVTs, increased expression of miR-517a/b and miR-517c results in decreased trophoblast invasion, increased production of the anti-angiogenic cytokine, TNFSF15, and increased production of sFLT1 but not VEGF or PLGF. In preeclampsia, both TNFSF15 and sFLT1 act as anti-angiogenic proteins contributing to placental dysfunction. This may provide insight into a possible functional mechanism for the actions of these miRNAs in the placenta. Collectively, these data not only give new insight into the level of gene regulation occurring within the placenta, but also, provides a plausible pathological mechanism for the role of these miRNAs in preeclampsia.

In recent years, the investigation into gene regulation by miRNAs in many pathological disease states including cancer, among many others, has increased significantly. However, research focused on the role of miRNAs in adverse obstetrical outcomes, including preeclampsia, is still in its infancy. A handful of studies have identified differences in both overall miRNA profiles and specific miRNAs in preeclamptic placentas when compared to normal term deliveries. Among them, two have identified miR-517 isoforms (miR-517c and miR-517*) as being altered in preeclamptic placentas using large scale high throughput miRNA arrays [28, 30]. Using real time QPCR to focus on miR-517a/b and miR-517c specifically, our study confirms that the expression of these miRNAs is increased in the preeclamptic placenta. Additionally, placentas from women with preterm preeclampsia, a more severe form of the disease, appear to have higher and less variable expression of both miR-517a/b and miR-517c suggesting that expression of these miRNAs may be associated with pathological mechanisms contributing to disease severity. Since it has been recently shown that miRNAs have the ability to be secreted from the placenta/trophoblasts via exosomes [23], it is interesting to note that miR-517 isoforms (miR-517a and miR-517c) have been found in the circulation of pregnant women [45, 46]. While the functional significance of miRNAs in the maternal circulation is unknown, the presence of miR-517 miRNAs in maternal blood may be simply a consequence of passive release from placental trophoblast or could suggest that these miRNAs may play a role in maternal-fetal communication or maternal adaptation to the fetus [47]. Additionally, it provides evidence for the possibility of miR-517 miRNAs to be used diagnostically as serum biomarkers of preeclampsia.

While the regulation of miRNA expression in the human placenta is not completely understood, several studies have found that oxygen tension within the placenta can have significant effects on miRNA expression [48, 49]. As the miR-517 family is most highly expressed in trophoblast cells [24, 30], we used primary first trimester EVTs to investigate hypoxia as a potential regulator of miR-517a/b and miR-517c expression. The expression of miR-517a/b and miR-517c was significantly increased by hypoxia suggesting that abnormal/pathological hypoxia, known to be associated with preeclampsia in early pregnancy, could be a significant regulator of trophoblast miR-517 expression. As there are no known studies to date that have shown hypoxic regulation of miR-517, we can only hypothesize that hypoxia-mediated changes in miR-517a/b and miR-517c are regulated by hypoxia- inducible transcription factors such as hypoxia inducible factor-1 alpha (HIF1-a), as has been shown for other hypoxia-mediated miRNAs such as miR-210 [50]. Interesting to note, the C19MC cluster of miRNAs (of which miR-517a/b/c are members) has been shown to be epigenetically regulated, primarily by DNA methylation. C19MC miRNAs are not readily detectable in most cell types outside of the placenta; however, when cancer cells are treated with demethylating agents, their expression is strongly increased [51]. A recent study, has shown that exposure to pathological hypoxia (<1% oxygen) alters the epigenetic profile in human trophoblasts [52]. Taken together, these data suggest that pathological placental hypoxia in early pregnancy could cause modifications of the epigenetic regulation of miR-517 miRNAs resulting in downstream altered trophoblast function.

miR-517a/b and miR-517c are highly expressed in the trophoblast cells of the placenta and are altered in preeclampsia, however, their role in regulating placental/trophoblast function is largely unknown. In this study, overexpression of miR-517a/b and miR-517c resulted in a highly significant decrease in trophoblast invasion suggesting that the increased miR-517a/b and miR-517c seen in preeclamptic placentas may target genes known to regulate trophoblast function early in pregnancy. While this is one of the first studies to attribute a function to the presence of miR-517a/b and miR-517c in the placenta and studies in other tissues/cell types are very limited, a recent study found that over expression of miR-517a and miR-517c inhibited cell proliferation of hepatocellular carcinoma cells due to G2/M cell cycle arrest through PYK2 (PTK2B), a predicted target of miR-517a/c [53]. Furthermore, they showed that blocking endogenous miR-517a and miR-517c resulted in aggressive cell growth and adhesion-dependent colony formation [53]. While not focused on miR-517a/b and miR-517c specifically, a recent study provided evidence that ectopic expression of C19MC miRNAs resulted in decreased trophoblast migration in HTR-8/SVneo cells [54]. Although invasion, migration and proliferation are separate and distinct cellular functions, all are vital for normal trophoblast function and may be regulated by miR-517 similarly. Of the 369 predicted targets (non-conserved) of miR-517a/b/c (TargetScan, www.targetscan.org), several genes including HOXA5 [55], SEMA3A [56], TFAP2B [57, 58] and PTK2B [59] have been shown to play a role in cellular invasion. Therefore, further studies investigating these direct downstream targets of miR-157a/b/c are necessary in order to fully understand the molecular mechanisms linking increased miR-517a/b/c to decreased invasion.

One of the most commonly accepted theories describing the pathogenesis of preeclampsia include the dysregulation or imbalance of placental angiogenic factors, including sFLT1, VEGF and PLGF, released into the maternal circulation and contributing to the maternal symptoms of this disease [60]. Additionally, sFLT1 expression has been shown to be significantly increased in the preeclamptic placenta [60, 61]. While many studies have investigated mediators of increased placental angiogenic factor production, including hypoxia, oxidative stress and genetic factors, a direct link is still unclear. In our study, overexpression of miR-517a/b and miR-517c in first trimester EVTs resulted in a significant increase in the release of sFLT1 with no changes in VEGF or PLGF suggesting that miR-517a/b and miR-517c may be altering the angiogenic balance in the placenta. The increase in sFLT1 would shift the balance towards an anti-angiogenic state contributing to the development of preeclampsia. Interestingly, overexpression of miR-517a/b and miR-517c resulted in an increase in sFLT1 instead of the expected gene repression seen with most miRNAs. This result would clearly suggest that sFLT1 is most likely not a direct target of miR-517a/b or miR-517c but may instead be secondarily regulated by other altered upstream targets. Additionally, neither VEGF nor PLGF are known direct or predicted targets of miR-517a/b or miR-517c, however, it is well known that a single miRNA has the ability to target multiple downstream transcripts and many transcripts can be regulated by multiple miRNAs amplifying the scope of putative miRNA gene regulation.

In an attempt to identify potential targets of miR-517a/b and miR-517c that may be regulating the observed increase in sFLT1, we focused on the TNFSF15 pathway. TNFSF15 (also known as VEGI) is a well-known cytokine that has been previously shown to activate the NF-kB and MAPK signaling pathways leading to increased apoptosis, an inhibition in endothelial cell proliferation and, most importantly for this study, a decrease in angiogenesis [62, 63]. In a recent study, TNFSF15 was identified as a regulator of VEGFR1 (FLT1) splicing and, hence, has the ability to alter the expression of sFLT1 [14]. In endothelial progenitor cells, increased TNFSF15, in association with the inhibition of JMJD6, resulted in an up-regulation of sFLT1 expression [14, 44]. Interestingly, we found that increased miR-517a/b and miR-517c expression resulted in an increased extravillous trophoblast expression of TNFSF15 but not JMJD6 or U2AF2, the gene encoding U2AF65, a known splicing factor regulated by JMJD6 [64]. Although TNFSF15 has been largely unstudied in the fields of pregnancy, preeclampsia and placental biology, these results provide some evidence that TNFSF15 may be contributing to the regulation of sFLT1 in trophoblast cells. While 1) TNFSF15 is mostly thought to be an endothelial cell secreted cytokine and 2) we found an increase in TNFSF15 and sFLT1 expression without a change in JMJD6, it is interesting to speculate that autocrine and/or paracrine effects between the trophoblast and vascular endothelial cells in the placenta may play a role in regulating sFLT1 release into the maternal circulation. Similarly to sFLT1, TNFSF15 does not seem to be a direct target of miR-517a/b or miR-517c due to an increase in gene expression as opposed to the expected gene repression seen with miRNAs. However, based on our previously published study[65], we hypothesize that NF-kB signaling could be a possible link between increased miR-517 and altered TNFSF15 expression. miR-517a and miR-517c activate NF-kB signaling through down regulation of TNFAIP3 interacting protein 1 (TNIP1), a direct miR-517a/b/c target. Interestingly, previous studies have shown that increased NF-kB signaling results in increased expression of TNFSF15, a secreted cytokine [66]. While further studies would be needed in order to clearly elucidate the role of NF-kB signaling in TNFSF15 expression, our results do provide evidence of a link between increased miR-517a/b/c in the preeclamptic placenta, activation of the TNFSF15 pathway and subsequent sFLT1 regulation within the trophoblast cells.

**Fig 6:**
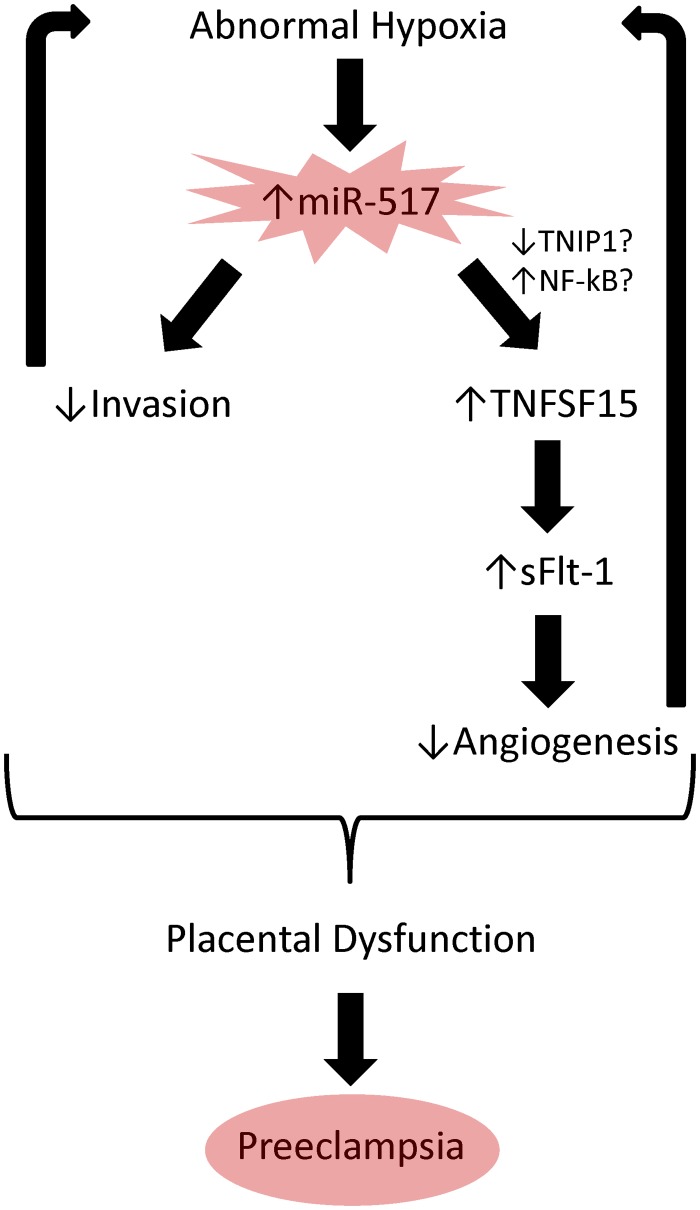
Schematic of hypothesized molecular pathways contributing to preeclampsia. Based on the results of this study, we provide evidence that abnormal pathologic placental hypoxia early in pregnancy could lead to increased miR-517a/b and miR-517c expression resulting in alterations in trophoblast function including 1) decreased trophoblast invasion and 2) increased TNFSF15 expression. Consequently, TNFSF15 is able to mediate FLT1 splicing and increase sFLT1 expression leading to a decrease in placental angiogenesis. Therefore, miR-517 mediated alterations in trophoblast function could propagate the placental hypoxia and contribute to the development of preeclampsia.

The results from this study provide evidence that miR-517a/b and miR-517c expression is dysregulated in the placenta of women with preeclampsia. Additionally, we have shown that these miRNAs are important regulators of placental function, specifically, increased expression of miR-517a/b and miR-517c results in decreased trophoblast invasion and increased TNFSF15 expression and sFLT1 release. This study not only provides further insight into the depth of molecular regulation occurring within the placenta during early pregnancy, but also suggests a possible functional role for miRNAs in regulating trophoblast function. As abnormal trophoblast function is a known pathological event associated with preeclampsia, altered placental expression of miR-517a/b/c, therefore, could potentially be a contributor to the development of this disease. Given the results of this study, it could be hypothesized that abnormal placental hypoxia resulting in an increased expression of miR-517a/b and miR-517c results in 1) decreased trophoblast invasion and 2) increased TNFSF15 which causes altered FLT1 splicing leading to increased sFLT1 release (Fig. 6). Further studies are warranted in order to clearly elucidate the direct downstream targets (possibly TNIP1 and NF-kB) regulating the miR-517-mediated alterations in placental function. However, the results of this study provide a clear starting point for future investigations into the role of these miRNAs in the pathogenesis of preeclampsia.
